# Frequent Detection of *KRAS*‐G12C and *PIK3CA*‐Q546K Mutations in MAP Tumors Highlights their Role in *MUTYH* Variants of Uncertain Significance Reclassification

**DOI:** 10.1155/humu/6589780

**Published:** 2026-05-18

**Authors:** Ana Beatriz Deleame Medeiros, Vanessa Nascimento Kozak, Clarissa Gondim Picanço-Albuquerque, Frederico Guilherme Keche Virmond Neto, Gabriel Oliveira dos Santos, Samuel Aguiar Junior, José Claudio Casali-da-Rocha, Dirce Maria Carraro, Giovana Tardin Torrezan

**Affiliations:** ^1^ Clinical and Functional Genomics, A.C.Camargo Cancer Center, São Paulo, Brazil, accamargo.org.br; ^2^ Department of Genetics, Federal University of Paraná (UFPR), Paraná, Brazil, ufpr.br; ^3^ Cancer Institute of Ceará, Haroldo Juaçaba Hospital, Fortaleza, Ceará, Brazil; ^4^ Virmond Medical Institute, Santa Tereza Hospital, Paraná, Brazil; ^5^ Department of Anatomic Pathology, A.C.Camargo Cancer Center, São Paulo, Brazil, accamargo.org.br; ^6^ Colorectal Tumors Reference Center, A.C.Camargo Cancer Center, São Paulo, Brazil, accamargo.org.br; ^7^ Oncogenetics Department, A.C.Camargo Cancer Center, São Paulo, Brazil, accamargo.org.br; ^8^ National Institute of Science and Technology in Oncogenomics and Therapeutic Innovation, São Paulo, Brazil

**Keywords:** *KRAS*-G12C, *MUTYH*-associated polyposis, *PIK3CA*-Q546K, variants of uncertain significance, VUS reclassification

## Abstract

Biallelic pathogenic variants in *MUTYH* cause *MUTYH*‐associated polyposis (MAP), a rare recessive colorectal cancer (CRC) predisposition syndrome characterized by somatic G:C > T:A transversions. The hotspot somatic mutations *KRAS*‐G12C and *PIK3CA*‐Q546K are highly enriched in MAP CRCs, and are rarely observed in sporadic cases, suggesting their potential utility in supporting the reclassification of variants of uncertain significance (VUS) in *MUTYH*. This study is aimed at evaluating the frequency of *KRAS*‐G12C and *PIK3CA*‐Q546K in adenomas and CRC from MAP patients and to demonstrate their relevance to reclassify *MUTYH* VUS. These hotspot mutations were evaluated using targeted NGS in adenomas and CRC tissues from 16 previously diagnosed MAP patients and three patients suspected of MAP, who harbored a VUS in either homozygosity or compound heterozygosity with a germline pathogenic *MUTYH* variant. *KRAS*‐G12C and *PIK3CA*‐Q546K were identified in 92.3% and 38.4% of 13 MAP adenocarcinomas, respectively. *KRAS*‐G12C was also present in 47% of 17 MAP adenomas, whereas none of them harbored *PIK3CA*‐Q546K. The detection of either mutation in CRC showed 100% sensitivity and 97% specificity for MAP (*p* = 0.00001). Among the three‐suspected MAP cases with VUS, two harbored somatic *KRAS*‐G12C and/or *PIK3CA*‐Q546K, providing sufficient evidence to reclassify *MUTYH* VUS p.Pro301Arg and p.Trp113Arg as likely pathogenic based on ACMG/AMP criteria. These findings support the use of *KRAS*‐G12C and *PIK3CA*‐Q546K as cost‐effective, accessible tumor biomarkers for aiding in MAP diagnosis and *MUTYH* VUS reclassification, particularly in settings with limited access to whole‐exome/genome mutational signature analysis.

## 1. Introduction

*MUTYH*‐associated polyposis (MAP) is an autosomal recessive condition with an estimated prevalence of 1:20.000–60.000 that predisposes individuals to colorectal adenomas and confers a high risk of colorectal cancer (CRC) [1, 2]. It is caused by biallelic germline pathogenic variants (GPV) in the *MUTYH* gene, which participates in the base excision repair pathway by excising adenines mispaired with 8‐oxo‐guanines [3]. When *MUTYH* is inactive, G:C > T:A transversions accumulate, affecting several CRC‐related genes, such as *KRAS*, *APC*, *PIK3CA*, and *SMAD4* [4, 5]. These transversions lead to characteristic mutational patterns known as single base substitution (SBS) signatures. In MAP tumors, SBS18 and SBS36 are the most prevalent, marked by an excess of C > A substitutions, reflecting oxidative damage and defective *MUTYH*‐mediated base excision repair, respectively [6].

In *KRAS*, G:C > T:A transversions generate the c.34G > T p.G12C somatic mutation, which is rare (<3%) in sporadic CRCs and other CRC‐predisposing syndromes, but highly frequent (>80%) in MAP tumors. A similar pattern is observed for *PIK3CA* c.1636C > A p.Q546K, reported in less than 1% in sporadic cases and > 20% of MAP tumors [4, 7–9]. Recently, Georgeson et al. [9] proposed the use of *KRAS*‐G12C and *PIK3CA*‐Q546K as supporting biomarkers for *MUTYH* variant classification, given their high frequency in MAP patients (84% in MAP vs. 2.4% in *MUTYH*‐negatives for *KRAS*‐G12C; 37% in MAP vs. 0.7% in *MUTYH*‐negatives for *PIK3CA*‐Q546K) and their association with SBS18 and SBS36 mutational signatures.

Germline variants in Mendelian cancer‐related genes are classified according to guidelines from the American College of Medical Genetics and Genomics and the Association for Molecular Pathology (ACMG/AMP) [10], as well as recommendations from the Clinical Genome Resource (ClinGen) [11] and the cancer predisposition gene variant database (CanVar‐UK) (https://canvaruk.org—last accessed on date: 20‐09‐2025). Variants of uncertain significance (VUS) lack sufficient evidence to be classified as either pathogenic or benign, and therefore have no defined clinical relevance. Tumor phenotypic features have been proposed as supportive evidence for pathogenicity [12]. Accordingly, the presence of *KRAS*‐G12C and *PIK3CA*‐Q546K in tumors from individuals suspected to have MAP may fulfill the ACMG/AMP PP4 criterion [10].

Here, we report the frequency of *KRAS*‐G12C and *PIK3CA*‐Q546K somatic mutations in adenomas and adenocarcinomas from individuals previously diagnosed with MAP. The high prevalence of these biomarkers in MAP CRC tumors supports their potential utility to reclassify VUS in *MUTYH*. We further applied this approach to three‐suspected MAP cases, leading to the proposed reclassification of two VUS as likely pathogenic.

## 2. Methodology

### 2.1. Study Cohort

**Figure 1:**
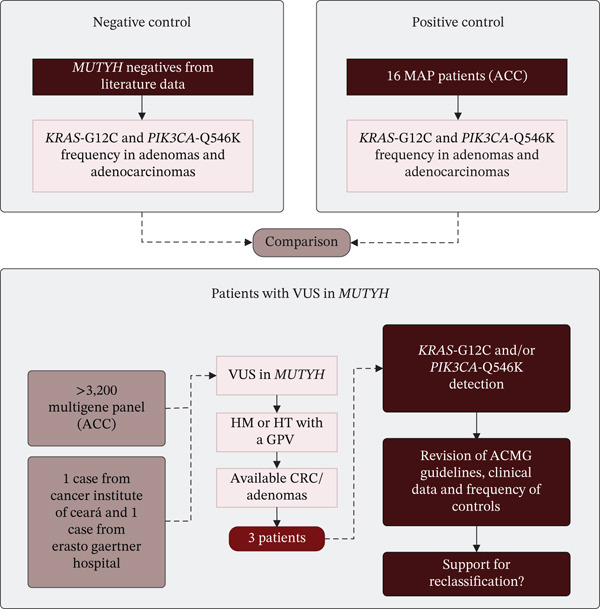
Overview of the methodological approach to reclassify VUS in *MUTYH* based on *KRAS*‐G12C and *PIK3CA*‐Q546K detection. Control groups were consisted of *MUTYH*‐negative patients from the literature [9, 13] (negative control) and MAP patients diagnosed at ACC (positive control), from whom we assessed *KRAS*‐G12C and *PIK3CA*‐Q546K detection frequency in adenomas and adenocarcinomas. Frequencies were compared across groups. For the test group, we analyzed individuals from a cohort of over 3200 germline tests performed at ACC and collaborating institutions (Cancer Institute of Ceará and Erasto Gaertner Hospital). Three patients were identified as carrying VUS in homozygosity or compound heterozygosity with available CRC and/or adenoma tissue samples. When either somatic hotspot mutation was detected, variant classification was re‐evaluated using ACMG/AMP/ClinGen guidelines, considering the patient′s clinical data and comparison with control frequencies, to determine whether sufficient evidence supported reclassification. Note: ACC = A.C.Camargo Cancer Center; VUS = variant of uncertain significance; HM = homozygous; HT = heterozygous; GPV = germline pathogenic variant; CRC = colorectal cancer; ACMG = American College of Medical Genetics and Genomics.

Patients over 18 years of age with CRC or polyposis and a previously identified VUS in the *MUTYH* gene, either in homozygosity or compound heterozygosity with a GPV, were selected from a cohort of over 3200 patients who underwent genetic testing for hereditary cancer predisposition syndromes at the A.C.Camargo Cancer Center (ACC), and from clinical testing cohorts of the Cancer Institute of Ceará and the Erasto Gaertner Hospital in Brazil. Additionally, patients over 18 years of age previously diagnosed with MAP at ACC were included as a positive control group. Formalin‐fixed paraffin‐embedded (FFPE) adenoma and/or adenocarcinoma tissue samples were retrieved from the Department of Pathology. All participants had provided informed consent, either through the institutional Biobank or via study‐specific consent forms. Clinical and histopathological data were obtained through review of electronic medical records and recorded in the REDCap database. This study was approved by the local ethics committees (CAAE 53067621.3.0000.5432). The negative control group (patients without GPVs in *MUTYH*) was obtained from the literature [9, 13] (Figure 1).

### 2.2. DNA Extraction

DNA was extracted from FFPE in the ACC Biobank following established standard operating protocols. DNA integrity was assessed with the TapeStation equipment (Agilent) and DNA concentration was determined using the Qubit equipment and the QUANT‐IT DNA HS assay kit (Life Technologies).

### 2.3. Multiplex PCR and Amplicon Sequencing

Targeted screening for *KRAS*‐G12C and *PIK3CA*‐Q546K somatic mutations was performed using a multiplex amplicon‐based next‐generation sequencing (NGS) approach. Primers targeting these regions were designed with Primer 3 software (Table S1). Multiplex PCR reactions were carried out with the Qiagen Multiplex PCR kit, using 20 ng of tumor DNA. The amplification protocol included an initial denaturation step at 95°C for 5 min, followed by 40 cycles of 30 s at 95°C (denaturation), 90 s at 60°C (annealing), and 30 s at 72°C (extension). Finally, a 10‐min extension step was carried out at 68°C. Amplification was confirmed by agarose gel electrophoresis, and the PCR products were then used for amplicon library preparation with the Ion Plus Fragment Library kit (Thermo Fisher). Libraries were sequenced on the Ion S5 platform (Thermo Fisher) according to the manufacturer′s instructions. Sequencing reads were analyzed using Torrent Suite software and Variant Caller (Thermo Fisher), and aligned with the GRCh37/Hg19 reference genome. Somatic mutations were reviewed using the Integrative Genomics Viewer Version 2.19.1 (IGV) and considered positive when present in ≥ 3% of total reads.

### 2.4. VUS Reclassification

VUS in *MUTYH* gene were reclassified according to the ACMG/AMP and ClinGen variant classification guidelines and updates. Based on the criteria met, variants were reclassified as pathogenic, likely pathogenic, benign, or likely benign. When somatic *KRAS*‐G12C and/or *PIK3CA*‐Q546K mutations were detected, the PP4 criterion was applied [10]. Variants with insufficient supporting evidence remained classified as VUS. All *MUTYH* variants were annotated using transcript NM_001128425.2.

### 2.5. Statistical Analysis

Inferential statistics were used to calculate the sensitivity, specificity, accuracy, positive predictive value (PPV), positive likelihood ratio (LR^+^), and negative likelihood ratio (LR^−^) of *KRAS*‐G12C and *PIK3CA*‐Q546K in indicating biallelic *MUTYH* carriers. Fisher′s exact test assessed the association for categorical data. A two‐tailed *p* value of less than 0.05 was considered statistically significant.

## 3. Results

### 3.1. Assessment of KRAS‐G12C and PIK3CA‐Q546K Somatic Mutations in MAP Adenomas and Adenocarcinomas

*KRAS*‐G12C and *PIK3CA*‐Q546K mutations were investigated in 17 adenomas and 13 adenocarcinomas from 16 MAP patients in the positive control group (Table S2). Twelve patients (75%) tested positive for *KRAS*‐G12C, and five (31.2%) were positive for *PIK3CA*‐Q546K. One patient harbored another pathogenic mutation in *PIK3CA* (c.1638G > T p.Q546H). When stratified by tissue type, *KRAS*‐G12C was found in 8/17 adenomas (47%) and in 12/13 adenocarcinomas (92.3%), whereas *PIK3CA*‐Q546K was detected only in adenocarcinomas (5/13, 38.4%). In comparison, literature data reported a frequency of 7.4% in adenomas and 2.4% in CRCs for *KRAS-*G12C, and 0.7% in CRCs for *PIK3CA-*Q546K (Table 1) in *MUTYH*‐negative patients [9, 13].

**Table 1:** Detection frequencies of *KRAS*‐G12C and *PIK3CA*‐Q546K in MAP adenomas and adenocarcinomas from our cohort and sporadic patients from the literature.

Somatic mutation	MAP data (present study)		Sporadic data	
			Walker et al. [13]	Georgeson et al. [9]
	Adenomas (*n* = 17)	Adenocarcinomas (*n* = 13)	Adenomas (*n* = 27)	Adenocarcinomas (*n* = 5364)
*KRAS*‐G12C	8 (47%)	12 (92.3%)	2 (7.4%)	127 (2.4%)
*PIK3CA*‐Q546K	Not detected	5 (38.4%)	Not detected	36 (0.7%)

Abbreviations: MAP, *MUTYH*‐associated polyposis; n, number of patients.

Detection of *KRAS*‐G12C in MAP CRCs was significantly higher when compared with *MUTYH*‐negative CRCs (*p* = 0.0001), with high sensitivity (92.3%), specificity (97.6%), accuracy (97.6%), and a LR^+^ of 38.99. Similar performance was observed when considering the presence of either *KRAS*‐G12C and/or *PIK3CA*‐Q546K (100%, 97%, 97%, and 33.11, respectively) (*p* = 0.0001). Testing only for *PIK3CA*‐Q546K in adenocarcinomas and *KRAS*‐G12C in adenomas showed limited sensitivity (38.4% and 47%, respectively), despite being significantly associated with MAP (*p* = 0.0001 and 0.0071, respectively). Sensitivity, specificity, and accuracy were not calculated for *PIK3CA*‐Q546K in adenomas, as this mutation was not detected in either the MAP or *MUTYH*‐negative control groups (Table 2).

**Table 2:** Statistical analysis of *KRAS*‐G12C and *PIK3CA*‐Q546K somatic mutations for detecting MAP patients.

	MAP	Non‐MAP ^∗^	Sensitivity	Specificity	Accuracy	PPV	LR^+^^∗^	LR^−^^∗^	*p*
**Adenocarcinomas**									
*KRAS*‐G12C positive	12	127	92.3%	97.6%	97.6%	10.5%	38.99	0.08	0.00001
*KRAS*‐G12C negative	1	5237							
*PIK3CA*‐Q546K positive	5	36	38.4%	99.3%	99.1%	14.7%	57.31	0.62	0.00001
*PIK3CA*‐Q546K negative	8	5328							
*KRAS*‐G12C and/or *PIK3CA*‐Q546K positives	13	162	100%	97%	97%	9.06%	33.11	0	0.00001
*KRAS*‐G12C and *PIK3CA*‐Q546K negatives	0	5202							
**Adenomas**									
*KRAS*‐G12C positive	8	2	47%	92.6%	92.4%	1.88%	6.35	0.57	0.0071
*KRAS*‐G12C negative	9	25							

*Note:* The asterisk ( ^∗^) denotes values from non‐MAP individuals and disease prevalence (0.3%) were obtained from Georgeson et al. [9].

Abbreviations: LR^+^, positive likelihood ratio; LR^−^, negative likelihood ratio; PPV, positive predictive value.

### 3.2. Reclassification of VUS in MUTYH

#### 3.2.1. Case 1

**Figure 2:**
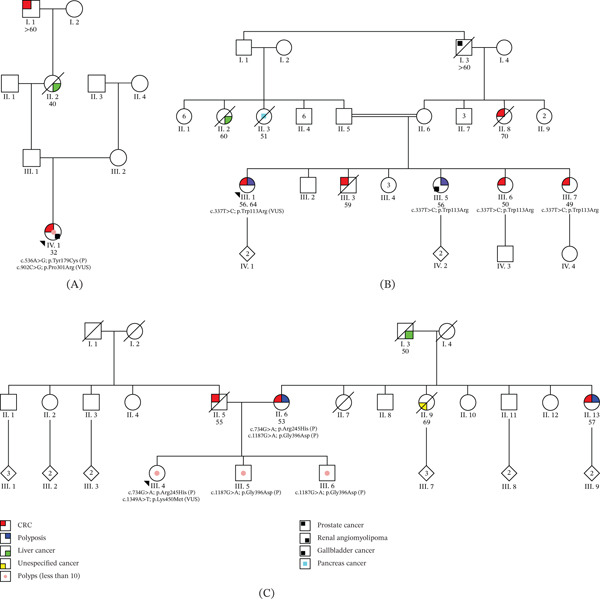
Pedigrees of probands carrying VUS in *MUTYH*. (A) Case 1: Proband carrying p.Try179Cys and p.Pro301Arg in compound heterozygosity. (B) Case 2: Proband carrying p.Trp113Arg (homozygous). (C) Case 3: Proband carrying p.Arg245His and p.Lys450Met in compound heterozygosity. Note: All family members tested are heterozygous for their variants, except for the sisters of proband B, who are homozygous for p.Trp113Arg. The numbers below each affected patient represent the age at cancer diagnosis. VUS = variant of uncertain significance; P = pathogenic; CRC = colorectal cancer.

A 32‐year‐old female was referred to a gastroenterologist after reporting hematochezia. Colonoscopy revealed the presence of pedunculated polyps (described in the clinical report as “more than one”) and a sessile polyp at the rectosigmoid junction, as well as a vegetative, infiltrative lesion in the distal rectum, subsequently identified as a poorly differentiated invasive adenocarcinoma. One year later, the proband was diagnosed with a renal angiomyolipoma. She reported a family history of cancer on her paternal side, including a great‐grandfather diagnosed with CRC over the age of 60 and a grandmother with liver cancer at the age of 40 (Figure 2A). Immunohistochemistry analysis showed preserved expression of mismatch repair proteins.

A germline multigene NGS panel (62 genes) identified two *MUTYH* variants: c.536A > G p.Tyr179Cys, heterozygous, classified as pathogenic (ClinVar Variation ID: 5293), and c.902C > G p.Pro301Arg, heterozygous, classified as VUS. The latter variant is absent in gnomAD [14] and ABraOm (a Brazilian populational database containing exome data of 1,171 healthy individuals) [15] and has not been reported in ClinVar or in the literature. Phase was confirmed as in trans using allele specific PCR [16].

Somatic analysis of the adenocarcinoma tissue revealed *KRAS*‐G12C (VAF: 9%) and *PIK3CA*‐Q546K (VAF: 8%). Based on ACMG criteria revision, *PM2_supp* (absence in population databases), *PP3_strong* (multiple lines of computational evidence support a deleterious effect on the gene—REVEL score: 0.96 [17] and *PM3* (for recessive disorders, detected in trans with a pathogenic variant) were applied. Additionally, *KRAS*‐G12C and *PIK3CA*‐Q546K detection supported the application of the *PP4* criterion and led to the reclassification of the p.Pro301Arg variant as likely pathogenic.

#### 3.2.2. Case 2

A female patient, born to consanguineous parents, underwent total proctocolectomy due to a personal history of polyposis and two metachronous CRC diagnosed at the ages of 56 and 64. Three siblings were also diagnosed with CRC at 49 (III.7), 50 (III.6), and 59 (III.3) years old (Figure 2B). Another sister (III.5) had polyposis and gallbladder cancer at 56 years old. Additional family history included maternal relatives (an aunt with CRC at age 70 and a grandfather with prostate cancer over age 60) and paternal relatives (two aunts diagnosed with cancer, one with liver cancer at 60 years old and the other with pancreatic cancer at age 51) (Figure 2B).

A multigene panel (31 genes) of the proband identified a homozygous variant in *MUTYH* (c.337 T > C p.Trp113Arg) classified as VUS by three independent ClinVar submissions (ID: 533303). This variant was previously reported in two compound heterozygous patients carrying the pathogenic p.Tyr179Cys variant: one diagnosed with polyposis and CRC at age 36 and another with polyposis [18]. Somatic analysis revealed the *KRAS*‐G12C mutation in an adenoma (VAF: 9%), and both *KRAS*‐G12C (VAF: 25%) and *PIK3CA*‐Q546K (VAF: 12%) in an adenocarcinoma. Later to the somatic analysis, targeted sequencing for the *MUTYH* variant was performed for the three sisters diagnosed with CRC or polyposis (III.5, III.6, and III.7), all three being identified as homozygous for the variant.

This variant is absent from gnomAD and ABraOm databases (*PM2_supp*), demonstrates a moderate deleterious effect in silico (REVEL score: 0.82—*PP3_moderate*), was detected in a homozygous state (*PM3_supp*), segregates with the disease (*PP1_strong*) [19] and was associated with *KRAS*‐G12C and *PIK3CA*‐Q546K (*PP4*). Collectivelly, the evidence supports reclassifying p.Trp113Arg as likely pathogenic.

#### 3.2.3. Case 3

A 46‐year‐old female sought genetic counseling due to polyps detected in recent colonoscopies and a family history of CRC. Her father was diagnosed with CRC and died at age 56, whereas her mother underwent a right colectomy at age 57 and later developed multiple polyps. The proband had three prior colonoscopies, revealing a total of five polyps (5–12 mm). No additional cancer cases were reported on her father′s side. On the maternal side, an aunt underwent total colectomy at 57 due to CRC and polyposis (> 10 polyps), and her maternal grandfather died in his fifties from liver cancer. Two younger brothers, under surveillance, had polyp burdens of seven and three, respectively (Figure 2C).

NGS multigene panel identified two heterozygous *MUTYH* variants: c.734G > A p.Arg245His (pathogenic, ClinVar ID: 140877) and c.1349A > T p.Lys450Met (VUS, ClinVar ID: 1041681). The p.Lys450Met variant is absent from population databases (*PM2_supp*) and had previously been observed in a polyposis patient in trans with a GPV (*PM3*). REVEL predicted a moderately deleterious effect (score = 0.81—*PP3_moderate*). The proband′s mother was later diagnosed with MAP as a compound heterozygote for two pathogenic variants (p.Arg245His and c.1187G > A p.Gly396Asp), and her brothers were heterozygous for p.Gly396Asp.

Somatic analysis of two low‐grade adenomas from the proband revealed *KRAS*‐G12V in one sample, with both adenomas negative for *KRAS*‐G12C and *PIK3CA*‐Q546K. Based on the combined findings, reclassification of the p.Lys450Met was not supported, and the variant remains classified as VUS.

## 4. Discussion

To our knowledge, this is the first study to evaluate the combined utility of *KRAS*‐G12C and *PIK3CA*‐Q546K somatic mutations for reclassifying VUS in the *MUTYH* gene. It is also the second to specifically investigate the relationship between the *PIK3CA*‐Q546K mutation and MAP, highlighting its high frequency in adenocarcinomas (38.4%; *p* = 0.00001) and absence in adenomas.

The high frequency of *KRAS*‐G12C in MAP tumors has been consistently reported since Lipton et al. [20] first described this mutation in 64% of adenocarcinomas and 43% of adenomas in MAP patients. Subsequent studies using advanced sequencing technologies reported even higher detection rates, reaching 70.5%–89% and 66.7%, respectively [4, 7, 9, 13]. Our findings showed *KRAS*‐G12C in 47% of adenomas and 92.3% of CRC from MAP patients, consistent with the literature and expected, as *KRAS* mutations commonly arise during the transition from early‐stage adenoma to more advanced stages [21].

By contrast, *PIK3CA*‐Q546K was just recently associated with MAP by Georgeson et al. [9], who reported its enrichment in CRCs (37%) compared with 0.7% in non‐MAP CRC cases. Previous studies detected this mutation in 0%–7.7% of adenomas and 20%–23.5% of MAP adenocarcinomas, supporting the hypothesis that it emerges later in CRC progression [4, 7, 13, 22]. In our study, testing for either *KRAS*‐G12C alone or both hotspots in adenocarcinomas provided high sensitivity (> 92%), specificity (> 96%), and accuracy (> 97%) for identifying MAP patients, with a strong LR^+^ (> 33).

Applying this approach to three individuals with family histories of CRC and polyposis, we reclassified two VUS (p.Pro301Arg and p.Trp113Arg) as likely pathogenic, both in individuals with CRC. For the third case, with the p.Lys450Met variant, evidence was insufficient to support reclassification, and further investigation of this VUS is warranted. This variant is located in the Nudix hydrolase domain, which is essential for *MUTYH*′s catalytic function [23], however, we did not apply the *PM1* criterion since it requires expert curation and supporting laboratory data to determine intolerant regions [24].

Although our findings, alongside prior evidence [9, 13, 25] support the use of *KRAS*‐G12C and *PIK3CA*‐Q546K as somatic biomarkers in MAP, certain limitations must be acknowledged. First, the ClinGen InSiGHT Hereditary Colorectal Cancer/Polyposis Expert Panel for *MUTYH* has not been officially published yet, as has been done for *APC*, for example [26]. Thus, the relative weight of specific ACMG/AMP criteria applied here in the reclassification of *MUTYH* VUS may still be provisional. Second, our study relied on literature‐based data for the negative control group, rather than analyzing a dedicated matched control cohort of *MUTYH*‐negative patients. Third, among the 17 adenomas tested, only one was classified as high‐grade; consequently, the actual frequency of *KRAS*‐G12C and *PIK3CA*‐Q546K in this tissue type may be higher in more advanced lesions.

Lastly, although the PPV for detecting MAP with either biomarker was modest (9.06%), this metric is inherently influenced by disease prevalence. Given the rarity of MAP, applying these markers to unselected CRC populations yields a lower PPV. However, evaluating multiple lesions—particularly high‐grade adenomas in combination with adenocarcinomas—and assessing additional somatic G > T transversions in genes such as *APC* may improve predictive value. Importantly, the high LR^+^ (LR^+^ > 33) and null LR^−^ observed in our cohort indicate that the detection of these hotspots strongly increases the posttest probability of MAP and provides substantial discriminatory power in clinically suspected cases.

VUS remain a frequent and challenging scenario in clinical practice, particularly when identified in genes that align with the patient′s or family′s phenotype. Tumor mutational signatures have proved valuable for VUS reclassification in other hereditary cancer syndromes, such as hereditary breast and ovarian cancer and constitutional mismatch repair deficiency [12]. Although tumor mutational signatures have also demonstrated high efficacy in identifying BER‐deficient tumors and hereditary CRC syndromes [9, 13], their implementation typically requires whole‐exome or whole‐genome sequencing, resources that remain cost‐prohibitive in many low‐ and middle‐income countries [27, 28]. Thus, cost‐effective biomarkers are essential to improve MAP diagnosis, as its variable phenotype and recessive inheritance frequently lead to misdiagnosis [5, 25]. In this context, tumor features are already being applied in variant interpretation frameworks. For instance, the InSiGHT group considers microsatellite instability and loss of mismatch repair protein expression in CRC and endometrial cancers as supportive evidence for reclassifying VUS in *MLH1*, *MSH2*, *MSH6*, and *PMS2* Lynch‐related genes. Similarly, since *KRAS* and *PIK3CA* are routinely tested in small NGS panels for prognostic and predictive purposes in CRC, especially for metastatic patients [29, 30], their broad availability and low cost make them practical tools to aid in the identification of MAP cases and to support reclassification of *MUTYH* VUS in clinical practice.

## 5. Conclusion

This study demonstrates the high prevalence of *KRAS*‐G12C and *PIK3CA*‐Q546K somatic mutations in adenocarcinomas from MAP patients. Detection of either mutation in colorectal tumors showed high sensitivity for identifying MAP cases and supports their use as accessible, cost‐effective biomarkers. Importantly, these findings enabled the reclassification of two *MUTYH* VUS (p.Pro301Arg and p.Trp113Arg) as likely pathogenic, underscoring the clinical utility of incorporating tumor somatic features into VUS interpretation frameworks in suspected MAP cases.

## Author Contributions

Conceptualization: G.T.T.; formal analysis: A.B.D.M. and G.T.T.; methodology: A.B.D.M.; investigation: A.B.D.M. and G.T.T.; funding acquisition: D.M.C. and G.T.T.; supervision: G.T.T.; Resources: V.N.K., F.G.K.V.N., C.G.P‐A., G.O.S., J.C.C.R., S.A.J., D.M.C., and G.T.T.; writing—original draft preparation: A.B.D.M., V.N.K., F.G.K.V.N., and G.T.T.; writing—review and editing: A.B.D.M., V.N.K., F.G.K.V.N., C.G.P‐A., G.O.S., J.C.C.R., S.A.J., D.M.C., and G.T.T.

## Funding

This study was supported by Collaborative Group of the Americas on Inherited Gastrointestinal Cancer (2022 CGA‐IGC Research Grant); Fundação de Amparo à Pesquisa do Estado de São Paulo (10.13039/501100001807, 2014/50943‐1 2022/05162‐8 2023/01303‐9); Conselho Nacional de Desenvolvimento Científico e Tecnológico (10.13039/501100003593, 465682/2014‐6); Coordenação de Aperfeiçoamento de Pessoal de Nível Superior (10.13039/501100002322, 88887.136405/2017‐00).

## Disclosure

All authors had access to the study data and had reviewed and approved the final manuscript.

## Conflicts of Interest

The authors declare no conflicts of interest.

## Supporting information


**Supporting Information** Additional supporting information can be found online in the Supporting Information section. Table S1: Primers and amplicon sizes employed in the multiplex PCR assay for the detection of somatic KRAS‐G12C and PIK3CA‐Q546K mutations. Table S2. Clinical features and information on the tissues evaluated in the 16 MAP patients comprising the positive control cohort.

## Data Availability

The most relevant data generated or analyzed during this study are included in this published article and its supporting information files. Additional information, including details on analytic methods or study materials, may be made available from the corresponding author upon reasonable request.
