# Supplementary material for: Frequent Detection of KRAS‐G12C and PIK3CA‐Q546K Mutations in MAP Tumors Highlights their Role in MUTYH Variants of Uncertain Significance Reclassification
Source: Hum Mutat. 2026 May 18;2026:6589780. doi: 10.1155/humu/6589780 (PMC13184164; doi:10.1155/humu/6589780)
Supplement: Supplementary file 1 — Supporting Information Additional supporting information can be found online in the Supporting Information section. Table S1: Primers and amplicon sizes employed in the multiplex PCR assay for the detection of somatic KRAS‐G12C and PIK3CA‐Q546K mutations. Table S2. Clinical features and information on the tissues evaluated in the 16 MAP patients comprising the positive control cohort. [file HUMU-2026-6589780-s001.pdf]

SUPPORTING INFORMATION

Table S1. Primers and amplicon sizes utilized in the multiplex PCR to screen for *KRAS*-G12C and *PIK3CA*-Q546K somatic mutations.

Somatic mutation	Primer forward	Primer reverse	Amplicon size (bp)
c.34G>T p.Gly12Cys (exon 2)	5’ AGGCCTGCTGAAAATGACTG 3’	5’ AGCTGTATCGTCAAGGCACTC 3’	81
c.1636C>A p.Gln546Lys (exon 10)	5’ CTCAAAGCAATTTCTACACGAG A 3’	5’ TGAGATCAGCCAAATTCAGTT 3’	148

Note: bp = Base pair. Transcripts: NM_004985.5 (*KRAS*); NM_006218.4 (*PIK3CA*).

Table S2. Clinical characteristics and information on the tissues assessed from the 16 MAP patients in the positive control group.

Patient	Age	Polyp burden	Family history of CRC/polyps	Family history of other cancers	*MUTYH* Variant (HGVS)	Adenomas			Adenocarcinomas	
						Grade	%TC	Result (VAF)	%TC	Result (VAF)
MAP_1	39	Multiple	Absent	Absent	c.1187G>A p.Gly396Asp / c.536A>G p.Tyr179Cys	Low	100%	*KRAS*-G12C (10%)	Not assessed	
						Low	50%	*KRAS*-G12C (10%)		
MAP_2	46	>40	CRC (sister, 57 yo)	Absent	c.536A>G p.Tyr179Cys / c.721C>T p.Arg241Trp	Low	80%	Negative	Not assessed	
						Low	10%	Negative		
						Low	10%	Negative		
MAP_3	34	>12	Not informed	Not informed	c.389-1G>C p. spl? / c.1147del p.Ala385fs	Low	50%	Negative	80%	*PIK3CA*-Q546K (18%)
						Low	40%	Negative		
MAP_4	52	Multiple	Not informed	Not informed	c.536A>G p.Tyr179Cys / c.389-1G>C p. spl?	Not assessed			80%	*KRAS*-G12C (20%); *PIK3CA*-Q546H (11%)
MAP_5	31	19	CRC + polyps (sister – 30 yo)	Absent	c.721C>T p.Arg241Trp[HM]	Low	20%	Negative	Not assessed	
						Low	80%	Negative		
MAP_6	31	1	CRC (brother – 26 yo)	Absent	c.536A>G p.Tyr179Cys / c.1227-1228dup p.Glu410Glyfs*43	Low	15%	Negative	Not assessed	
MAP_7	52	Multiple	Absent	Gynecological cancer (mother – 73 yo); Leukemia (paternal aunt)	c.536A>G p.Tyr179Cys / c.1147del p.Ala385fs	High	80%	*KRAS*-G12C (26%)	Not assessed	
MAP_8	44	>1	Polyposis (sisters)	Absent	c.348+33_*64+146del4285 p.spl? [HM]	Low	50%	*KRAS*-G12C (25%)	80%	*KRAS*-G12C (30%)
MAP_9	58	16	CRC (sister 1 – 58 yo)	Endometrial cancer (sister 2 <50 yo);	c.536A>G p.Tyr179Cys / c.325C>G p.Arg109Gly	Low	50%	*KRAS*-G12C (29%)	NA	*KRAS*-G12C (NA)
MAP_10	27	Absent	CRC (father – 61yo, 2 paternal uncles)	Absent	c.348+33_*64+146del4285 p.spl? [HM]	Not assessed			NA	*KRAS*-G12C (NA); *PIK3CA*-Q546K (33%)

MAP_11	56	>12	Absent	Absent	c.536A>G p.Tyr179Cys / c.721C>T p.Arg241Trp	Low	60%	*KRAS*-G12C (6%)	50%	*KRAS*-G12C (NA)
MAP_12	63	Not informed	Not informed	Not informed	c.1187G>A p.Gly396Asp / c.721C>T p.Arg241Trp	Not assessed			60%	*KRAS*-G12C (NA); *PIK3CA*-Q546K (4%)
MAP_13	44	>1	CRC (brother)	Stomach cancer (mother, brother); Larynx cancer (father); CNS cancer (brother)	c.348+33_*64+146del4285 p.spl? [HM]	Low	90%	*KRAS*-G12C (35%)	60%	*KRAS*-G12C (11%)
						Low	40%	*KRAS*-G12C (15%)	50%	*KRAS*-G12C (6%); *PIK3CA*-Q546K (8%)
MAP_14	59	Absent	Not informed	Not informed	c.1187G>A p.Gly396Asp / c.536A>G p.Tyr179Cys	Not assessed			70%	*KRAS*-G12C (49%)
MAP_15	50	34	Absent	"Throat" cancer (paternal uncle)	c.1187G>A p.Gly396Asp / c.933+3A>C p.spl?	Low	40%	Negative	80%	*KRAS*-G12C (43%)
MAP_16*	32	>1	CRC (paternal great-grandfather >60 yo)	Liver cancer (paternal grandmother – 40 yo)	c.536A>G p.Tyr179Cys / c.902C>G p.Pro301Arg	Not assessed			40%	*KRAS*-G12C (9%); *PIK3CA*-Q546K (8%)

Note: *Patient described in the article (reclassification of the p.Pro301Arg VUS as likely pathogenic). The age reported in the table refers to the age of CRC onset. For patients who did not develop a tumor, the age at first polyp onset was considered. HM = Homozygous; HT = Heterozygous; VAF = Variant allele frequency; NA = Not available; TC = Tumor cells.
